# Ethical, medico-legal and financial aspects of the legalization of surrogate maternity in Bulgaria

**DOI:** 10.4314/ahs.v24i4.19

**Published:** 2024-12

**Authors:** A Mihaylova, D Bakova, D Davcheva, A Yaneva, D Shopova, S Harizanova, N Parahuleva, M Semerdzhieva

**Affiliations:** 1 Department of Health Care Management, Faculty of Public Health, Medical University of Plovdiv, Bulgaria; 2 Department of Clinical Laboratory, Faculty of Pharmacy, Medical University of Plovdiv, Bulgaria; 3 Department of Medical Informatics, Biostatistics and E-learning, Faculty of Public Health, Medical University of Plovdiv, Bulgaria; 4 Department of Prosthetic Dentistry, Faculty of Dental Medicine, Medical University of Plovdiv, Bulgaria; 5 Department of Hygiene, Faculty of Public Health, Medical University of Plovdiv, Bulgaria; 6 Department of Obstetrics and Gynaecology, Medical University of Plovdiv, Plovdiv, Bulgaria

**Keywords:** Surrogate maternity, reproductive ethics, law regulation

## Abstract

The legalization of surrogacy and reproductive choices is a possible solution for childless families and the specialists in this area prove that this is a great challenge and responsibility. There are many issues on the topic: medical, ethical, as well as socio-psychological, legal and financial. The aim of present survey is to study and analyse the attitudes of society regarding some medical, social and economic and law aspects related to the legalization of surrogate maternity in Bulgaria. An anonymous survey was conducted involving 387 people. The data was collected via a pen and paper questionnaire pack. The initiative has provoked wide public interest. According to the participants in the present study, in the event of it being legalized, surrogate maternity should not become a profession, a source of surplus income for the surrogate mother, but the costs directly related to the pregnancy, birth and postpartum recovery should be compensated. The rights of the surrogate mother regarding custody of the child after birth and abortion rights should be limited in favor of the intended parents. The aspects discussed concern only the projected legal framework of surrogate maternity in Bulgaria, because at the present, the Bill has not been adopted.

## Introduction

Surrogacy is a topic that raises many questions and discussions, mostly from ethical and moral point of view. In many countries, surrogacy is completely legal and regulated by laws and regulations, in other countries it is banned, and in some countries, there is no specific solution to the issue. There is no special law in Bulgaria, but the ban is contained in an ordinance issued in 2007 by the Minister of Health, which has an annex that specifies the medical standard “Assisted Reproduction” where it is written that surrogate pregnancies are not allowed. In this legal definition^1^ surrogacy is a method whereby a woman wears pregnancy instead of another and after the birth of the child she surrenders it to the biological parents. Either the surrogate mother provides her womb for the wearing of another genetic material, and after childbirth there are no rights to the child. Many families and couples who, for medical or other reasons, cannot have children, are looking for alternatives to create their own generation. At a time of demographic crisis, surrogacy could be a good alternative. The main argument of the initiators of the bill in Bulgaria is that legal regulation is a kind of prevention of the risks that exist for families hoping for a child as well as the regulation of the status of the surrogate mother. In some countries, people support surrogacy, but mostly for medical reasons, while others give support when it comes to non-commercial surrogacy. The upcoming legislative changes in Bulgaria have prompted our interest in exploring public opinion related to surrogacy. Modern medicine, specifically assisted reproductive technology (ART), has overtaken the law in many jurisdictions around the world^2^. Countries around the world have responded to the emergence of assisted reproductive technologies (ART) in a variety of ways. Some countries prohibit certain treatment, others support them through health insurance, while other countries permit them but do not help pay for them. Because of these differences, not all people have the same degree of access to ART^3^. In recent years, there has been a revival of interest in the procedure of using a surrogate mother to help infertile couples have a child. On the one hand, it is the purely human desire of socially suited families to have a child through morally acceptable methods. On the other hand, the question of the potential risks involved in this long-term solution stand. The ethical aspects of surrogacy set a number of requirements for a strict international legal regulation of this socially sensitive area of human reproduction, in full consonance with local culture, traditions and attitudes of public opinion. Some groups condemn ART and want it banned, while its supporters acknowledge the need for legislative guidelines and regulations. If surrogacy takes place in an accepting society, it is possible that surrogates may be better supported and less stigmatized^4^. Legal restrictions, cultural differences, religious beliefs, and variation in access to advanced reproductive technologies (ART) contribute to the rise in cross-border reproductive care (CBRC)^5^. The lack of legal regulation, including Bulgaria, favors the development of transnational surrogacy^6^,^7^. Regarding the legalization of surrogate maternity, five Bills have been submitted in the Parliament of the Republic of Bulgaria^8^. The amendments adopted at first reading by the 41st National Assembly to resolve surrogate maternity in Bulgaria were not put to the vote at second reading. However, the Bulgarian society continues to be interested in the issue. The aim of present survey is to study and analyse the attitudes of society regarding some ethical, social and economic aspects related to the legalization of surrogate maternity in Bulgaria.

## Material and Methods

For the purpose of the study are used sociological methods – questionnaire survey and statistical methods – statistical analysis, descriptive statistics, chi-squared test. An anonymous survey was conducted involving 387 individuals, aged 20 to 61 years (mean 37.28 ± 0.54), of which 48.68% were men and 51.32% women. High school graduates were the largest group of respondents (81.13%). The data was collected via a pen and paper questionnaire pack. It was then returned anonymously to the authors.

### Statistical analysis

The statistical evaluation was completed using the Statistical Package for the Social Sciences for Windows, version 19.00. The descriptive statistics were presented as frequency, proportion, and mean ± standard deviation. A chi-squared test was used to evaluate the significance of the difference. A value of p<0.05 was considered significant.

## Results and Discussion

### Compensation of surrogate maternity

Regarding the question of whether surrogate maternity should be accompanied by payment to the surrogate mother, without mention of whether these costs should be borne by the state or intended parents, 66.41% of the respondents are of the opinion that there should be compensation. Of these, 25.28% consider extra compensation, in excess of direct costs, to be acceptable. Although none of the existing legislations authorizing surrogate maternity perceive it as a completely free service, 17.74% of our respondents advocate this idea (Table 1). There was a significant difference in opinion between the different genders (p<0.01, χ2 = 13.74). Women believe that all costs of the mother during the pregnancy should be compensated, while men, unlike women, often think that additional compensation should be paid, or the service should be free of charge.

**Table 1:** Opinions of the respondents regarding compensation of surrogate maternity

Compensation of surrogate maternity	Absolute number	%
Compensation of only relevant expenses (directly related to the cost of bearing the child) should be included	129	50.39
Compensation for loss of potential earnings (the surrogate mother does not work during the pregnancy) should be included	73	28.51
No Compensation	35	13.68
No Opinion	19	7.42
Total	387/256	100.0

### Surrogate mother's right to custody of the child

We raised the question of the surrogate mother's rights with respect to custody of the child. Only 8.30% of respondents support the moral right of the surrogate mother to change her mind during her pregnancy, as a result of inability to cope with the strong emotional attachment to the child she has carried for nine months. A significant part of respondents (72.08%) considered this issue as an item of the surrogacy contract, which must be respected.

### Surrogate mother's right to interruption of pregnancy (abortion)

**Figure 1:**
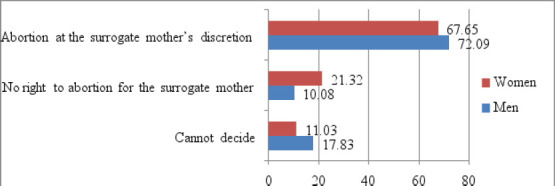
Opinions of the respondents regarding the surrogate mother's right to abortion

Regarding the issue of the surrogate mother's autonomy in abortion decisions for various reasons, we received interesting results. According to 69.81% of respondents, it is permissible to interrupt the pregnancy not only due to medical reasons but also at the surrogate mother's discretion. Only 14.34% cannot decide (Figure 1). Men are more definite in their opinion that the surrogate mother should not be contractually obliged to carry the baby to birth, if circumstances requiring abortion arise during the pregnancy (p<0.05, χ2 = 7.61). The possible implications of such an option for extending the rights of the surrogate mother as a possibility for extortion of the family, benefiting from its services, should be discussed. (Figure 1)

### Responsibility for the child

The survey covered the question of cases where the child produced through surrogacy was abandoned. According to the present study, the responsibility should be borne by the couple (61.13%), on whose initiative it was conceived. The next most frequently given response was that responsibility should be taken by the respective government institutions. Only 10.57% believe that the surrogate mother should take care of the child. As a possible cause of reluctance on both sides of the contract for responsibility for a carried child, cases of children born with malformations are most often considered. There was a significant difference in respondents' opinion according to their age (p<0.05, χ2 = 19.88). Older participants (over 35) are of the opinion that responsibility for raising the newborn, when the intended parents and the surrogate mother decline, should be given to the intended parents, while younger participants (under 25) believe that government institutions should have the responsibility. Although it cannot be said that surrogate maternity is common in Bulgaria, which is not yet legal, there is still a real threat of abuse in this direction^9^.

The question of whether it is ethical to compensate surrogate mothers has caused concern and debate over the years. In the USA, payment is permitted and covered by contracts. In the UK and European countries which allow surrogacy, the law permits only altruistic surrogacy. The European Society of Human Reproduction and Embryology's (ESHRE) Task Force on Ethics and Law states that “payment for surrogacy services is unacceptable” 10,11. The latest International Federation of Fertility Societies Surveillance Report 2013 states only that “payment of surrogate host is reported as continuing to be an issue that provokes many debates”^12^. The present legislation in Bulgaria defines the mother of the child as the woman which has given birth. This is a cause of serious concern to intended parents, as they may not receive custody of the child. Another serious problem that may arise is the surrogate mother's desire to keep the baby. There have been cases of surrogates who became emotionally attached, changed their minds either during pregnancy or after birth and decided to keep the child. In their study, Shayestefar M. and Abedi H. found that motherhood love and affection arises during pregnancy and progresses^13^. In this regard, there are some concerns that can be removed through careful ratification, surrogacy contract and two sides' awareness. According to the current study and the Krastev online survey, about 70% of the Bulgarian community finds it unacceptable for the child to be kept by the surrogate mother^14^. In most countries where surrogacy is permitted, a surrogate mother has to decide whether to retain or surrender the child. An ideal surrogate should be able to avoid forming any attachment with the child that negates the surrogacy arrangement. Of interest is the debate on the right of intended parents to control the pregnancy, require an additional prenatal diagnosis and insist on abortion at their discretion. An important point in the surrogacy contract is the degree of eligibility to exercise control over lifestyle, nutrition, physical activity and harmful habits during pregnancy in order to reduce the risk of fetal harm. Lifestyle, unhealthy behavior, harmful habits, exposure to polluted environment such as heavy metals can be associated with congenital malformations incompatible with life, causing termination of pregnancy^15^. The experience of Dar S. et al. in Canada suggests a strict program, a clear and transparent process and tight collaboration between medical, legal and social professionals which have guaranteed success in the vast majority of cases^16^. Serious preventive measures are needed to reduce the risks to children produced through surrogacy^17^,^18^. This will significantly reduce te number of children with disabilities and the number of abandoned children. In this regard, further education for professionals that could care for surrogate mothers is certainly needed. The recommendations of the Practice Committee and the Ethics Committee of the American Society of Assisted Reproduction (ASRM) are set out in detail in their most recent reports and are well worth reading in full by professionals practicing or intending to practice treatment of surrogacy^19^. In order to prevent the use of genetically damaged material, the Practice Committee report sets out “guidelines for the screening and testing of genetic parents and gestational carriers to reduce the possibility of complications and to address the complex medical and psychological issues that confront the gestational carriers and intended parents, as well as the children” 20.

Aside from the personal difficulties of childless families, the legislator will have to take responsibility for the social consequences of legalizing surrogate maternity. An important problem could arise if prospective parents give up the child for various reasons – separation of partners, death of one of the partners, birth of a disabled child, etc.^21^. In such cases, the child is the most vulnerable party among all involved^13^. The strength of the present study is the use of a non-representative, self-selected sample, which did not report reproductive problems. The limitation is that the discussed aspects refer only to the projected legal regulation on surrogacy arrangements in Bulgaria, since the Bill has not been adopted by the National Assembly to date. Legislators may amend national laws to remedy at least the most typical situations that occur. In any event, the harmonization of national rules regarding the most important points is necessary.

## Conclusions

The contemporary forms of medical intervention in the reproductive capacity of people have become widespread over the last decades and are occurring against the backdrop of fundamental changes in moral judgment and legal status.

The initiative to legalize surrogate maternity in Bulgaria has provoked wide public interest. According to the participants in the present study, in the event of it being legalized, surrogate maternity should not become a profession, a source of surplus income for the surrogate mother, but the costs directly related to the pregnancy, birth and postpartum recovery should be compensated. The rights of the surrogate mother regarding custody of the child after birth and abortion rights should be limited in favor of the intended parents. The aspects discussed concern only the projected legal framework of surrogate maternity in Bulgaria, because at the present, the Bill has not been adopted by the National Assembly of the Republic of Bulgaria.

It is necessary for the public to be sufficiently informed, both in order to be able to participate openly in the discussion and to be ready to tolerate the beneficiary families and problems associated with the procedure. The discussion regarding the legalization of surrogate maternity is forthcoming. There are still many unclear moments, critical points and risk of insufficient regulation. Professionals will have to support their decision with sufficient theoretical experience and high responsibility, and overcome the gaps and lack of guarantees regarding the ethical, legal, and social consequences of the legalization of surrogate maternity.
